# Randomised Controlled Trial Comparing Daily Versus Depot Vitamin D3 Therapy in 0–16-Year-Old Newly Settled Refugees in Western Australia Over a Period of 40 Weeks

**DOI:** 10.3390/nu10030348

**Published:** 2018-03-13

**Authors:** Ushma Wadia, Wayne Soon, Paola Chivers, Aesen Thambiran, David Burgner, Sarah Cherian, Aris Siafarikas

**Affiliations:** 1 Department of Rheumatology and Metabolic Medicine, Princess Margaret Hospital for Children, Perth, WA 6008, Australia; Ushma.Wadia@health.wa.gov.au; 2 Department of Infectious Diseases, Princess Margaret Hospital for Children, Perth, WA 6008, Australia; 3 Division of Paediatrics, School of Medicine, Faculty of Health and Medical Sciences, The University of Western Australia, Perth, WA 6008, Australia; Wayne.Soon@health.wa.gov.au (W.S.); sarah.cherian@health.wa.gov.au (S.C.); 4 Institute for Health Research, The University of Notre Dame Australia, Fremantle, WA 6160, Australia; paola.chivers@nd.edu.au; 5 School of Medical and Health Sciences & Exercise Medicine Research Institute, Edith Cowan University, Perth, WA 6027, Australia; 6 Western Australian Bone Research Collaboration, Perth, WA 6008, Australia; 7 Humanitarian Entrant Health Service, North Metropolitan Health Service, Perth, WA 6000, Australia; Aesen.Thambiran@health.wa.gov.au; 8 Department of Paediatrics, The University of Melbourne, Melbourne, VIC 3052, Australia; david.burgner@mcri.edu.au; 9 Department of Paediatrics, Monash University, Melbourne, VIC 3168, Australia; 10 Murdoch Children’s Research Institute, Royal Children’s Hospital Melbourne, Melbourne, VIC 3052, Australia; 11 Refugee Health Service, Department of Paediatrics, Princess Margaret Hospital for Children, Perth, WA 6008, Australia; 12 Department of Endocrinology and Diabetes, Princess Margaret Hospital for Children, Perth, WA 6008, Australia

**Keywords:** depot, daily, supplementation, therapy, vitamin D, refugee, rickets

## Abstract

Vitamin D deficiency is highly prevalent in newly settled refugees in Western Australia (WA). If adherence to daily vitamin D therapy is problematic, depot therapy is a therapeutic alternative. The aim of this study was to compare daily versus depot treatment and factors influencing the therapeutic outcome. Newly settled refugees (*n* = 151) with 25(OH)D levels less than 78 nmol/L were randomised to receive daily or depot vitamin D therapy with eight weekly interval follow up to 40 weeks. Biochemical and clinical parameters were collected at each visit. Generalized Linear Mixed Models (GLMM) examined the longitudinal changes over time controlling for confounders including age, gender, treatment arm, season, country of refuge/origin and sun exposure score. Participants were aged 5.5 months to 16.0 years (75 males, 83 females). Both treatment groups achieved vitamin D sufficiency. The daily treatment group had significantly higher 25(OH)D levels at each visit post baseline and a higher proportion of participants with levels above 50 nmol/L at all time points. Time, treatment group, calcium and sun exposure score were significant predictors of 25(OH)D serum levels. Depot vitamin D therapy is an alternative to daily treatment in this at-risk group of children and adolescents in whom treatment adherence is problematic.

## 1. Introduction

Vitamin D deficiency is one of the most common nutritional deficiencies in the world with approximately one billion people at risk [1,2]. It is on the rise in developed countries, even in Australia where there is an abundance of sunlight [3]. Additional risk factors among newly settled refugees include veiling, dietary deficiencies, darker skin colour and unfamiliarity with the local healthcare system [4,5]. More specifically, in Australian children with vitamin D deficiency rickets, risk factors include dark skin and maternal veiling with 96–98% of these being children migrants or born to a migrant parent [6,7].

Vitamin D is involved in the regulation of several skeletal and non-skeletal functions through the action of its active metabolite, 1,25(OH)_2_D. Research into non-skeletal effects of vitamin D has increased greatly in recent years [8,9]. Some of these associations include suppression of proliferation and differentiation of cancer cells, modulation of innate and adaptive immunity, modulation of muscle cell proliferation, improved cardiovascular health, modulation of pancreatic beta cell function and insulin sensitivity, clearance of amyloid plaques and promotion of survival, development and neuron function [9,10,11,12,13]. Furthermore low vitamin D concentrations have been associated with increased longitudinal risk of hypertension, diabetes, cardiovascular disease and atherosclerosis [11].

Vitamin D deficiency, whilst prevalent, is often asymptomatic. Clinical features include rickets, craniotabes, bone pain, muscle pain, hypocalcaemia seizures, delayed gross motor milestones and irritability [14], but more likely in infants compared to older children and adolescents.

Increasing sunlight exposure within recommendations for the prevention of skin cancer is the ideal method of improving vitamin D status, but it may not be suitable or effective for refugee children due to darker skin colour, veiling and other socio-cultural factors [15,16,17]. A recent international consensus paper recommends implementing national supplementation and fortification of food programmes with vitamin D and/or calcium to address the high rates of nutritional deficiency [2,18]. For high risk ethnic groups, vitamin D supplementation during every winter and spring has been suggested [2].

Due to the longer periods of treatment required in this group, adherence with daily oral vitamin D supplementation is problematic and hence, depot (or “stoss”) vitamin D supplementation at larger doses in intervals of weeks or months may be a suitable alternative therapeutic option [2,19]. The use of high-dose depot vitamin D therapy is increasing, but there are little data on its use in children. In Australia, the experience with depot vitamin D therapy is limited.

In Western Australia (WA), the Humanitarian Entrant Health Service (HEHS) provides health assessments for all refugee and humanitarian entrants who have been resettled in the state under the Humanitarian Programs. Vitamin D assessment is conducted for all refugees who access this service. At the time of the study approximately10,000 refugees were resettled in Australia annually, with over 10% resettled in WA [20]. Children represent about half of the resettled refugees. The main source regions for offshore refugee and humanitarian visas during the study period were Middle East, South West Asia and Africa [20]. Vitamin D deficiency remains a common referral reason to the Princess Margaret Hospital for Children (PMH) Refugee Health Service (RHS) [21].

The aim of this study was to compare the efficacy of daily and depot vitamin D therapy in newly settled refugees aged 0–16 years with low vitamin D levels and to analyse factors influencing the therapeutic outcome.

## 2. Materials and Methods

### 2.1. Trial Design

This is a prospective randomised controlled trial, approved by the PMH Human Research Ethics Committee (registration number 1564/EP), and registered with the World Health Organisation (WHO, # U1111-1125-4879) and Australian and New Zealand Clinical Trials Registry (http://www.anzctr.org.au/; ANZCTR, ACTRN12611001177943). Inclusion criteria were children aged 0–16 years, refugee background and a 25(OH)D level lower than 78 nmol/L. Participants were excluded if they were already on Vitamin D supplements. Professional interpreters were used for the duration of the study in keeping with the WA Department of Health Language Services Policy.

In this context, vitamin D deficiency is commonly thought of as a spectrum, from optimal vitamin D status to insufficiency to deficiency. There is ongoing debate about what the optimal serum 25(OH)D thresholds should be. Levels <50 nmol/L are widely regarded as vitamin D deficient by some groups, and a target of ≥50 nmol/L is currently recommended for infants, children, and adolescents in Australia [2,14,22,23,24]. For adults >75 nmol/L has been proposed as the level needed to support extra-skeletal functions of vitamin D [1,25]. This has not been confirmed for children [14,26]. We used 78 nmol/L as the cut-off for vitamin D insufficiency and 27.5 nmol/L for vitamin D deficiency based on the level of 25(OH)D that induces a positive PTH-response to maintain normocalcaemia [27].

### 2.2. Participants

Participants were recruited from HEHS, where they were screened for their eligibility during initial health assessment. Written informed consent was obtained from primary caregivers and interpreters utilised for all study related activities including explanation of the study, consent, plan for follow up, administration of questionnaires and treatment plan where indicated.

### 2.3. Intervention

A concentrated water-soluble emulsified vitamin D3 solution was used for daily therapy (Bio-Logical Vitamin D3 solution at 5000 IU/mL, Biological Therapies, Braeside, VIC, Australia), and a 50,000 IU/mL vitamin D3 solution in olive oil prepared by the PMH Clinical Trials Pharmacy was used for depot therapy. Calcium supplements (200–600 mg elemental calcium per day) were also prescribed for participants whose diet was low in calcium and those with hypocalcaemia (<2.15 mmol/L).

### 2.4. Outcomes

The following were recorded at the visits:

(i) Clinical parameters: height, weight, clinical signs of rickets (bone deformities, widened epiphyses, craniotabes and rachitic rosary);
(ii) Biochemical parameters: serum 25(OH)D (DiaSorin Liaison, DiaSorin (PTY) LTD, NSW, Australia), calcium and alkaline phosphatase (ALP) (Abbott Architect, Abbott Diagnostics Division, NSW, Australia or on Vitros 250, Ortho Clinical Diagnostics Australia, VIC, Australia);
(iii) A sun exposure questionnaire was applied using the recall method (duration and timing of sun exposure four days before the visit, factors affecting sun exposure including weather, season, clothing, hat and sun screen). This information was transformed into a validated score system “sun exposure score (SES)” based on a method by Specker et al. [28]; with higher scores equivalent to greater sun exposure;
(iv) Nutrition was assessed using a three-day food diary. This information was analysed using software of the German Society for Nutrition (DGE-PC Professional Version 2.8.0.26, 2007) to assess average dietary calcium and vitamin D intake across the three major ethnic groups: Asian, Middle-Eastern and African;
(v) Data on country of origin and country of refuge (last country of transit) were included.

### 2.5. Sample Size

Power calculation determined eight participants (*n* = 8) per group were needed to detect a change of 5 nmol/L in 25(OH)D levels with power set at 0.8 and alpha of 0.05.

### 2.6. Randomisation

**Figure 1:**
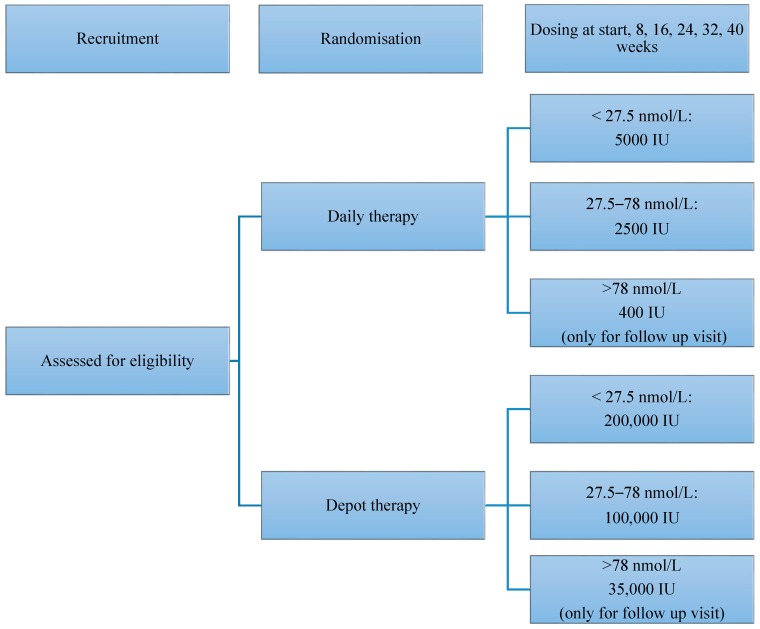
Study design.

Participants were randomised to receive either daily or depot vitamin D therapy by selecting a number between one and 10 from a sealed opaque envelope (odd numbers assigned to the daily group, even numbers to the depot group). However, if multiple children from the same family were recruited, each were assigned to the same treatment group. Participants remained in the same treatment group for the duration of the study. Follow up appointments were every eight weeks for up to 40 weeks and included a clinical examination, blood test, administration of sun exposure and dietary questionnaires and supply of medication. Depot vitamin D was administered under supervision at the time of the visit. Doses were standardised according to serum 25(OH)D levels at the time of recruitment and subsequent follow up (Figure 1). The total depot dose was slightly lower than the sum of the daily dose over the eight-week period of therapy.

For the first 50 participants, they exited the study if the 25(OH)D was >78 nmol/L and if the level was still low, they had a planned RHS review. Partway, a protocol amendment was incorporated following interim analysis to allow optimal longitudinal follow up data to 40 weeks. Subsequently, participants exited the study if there was no longer need for further follow up at the RHS and they had achieved levels of >78 nmol/L.

Parents or guardians were contacted a week before the appointment, using telephone interpreters, to minimise participant loss.

### 2.7. Statistical Methods

Statistical analysis was performed using the Statistical Package for Social Sciences (SPSS) software (version 24.0, IBM Corporation, New York, NY, USA). Four additional variables were calculated; 25(OH)D levels were dichotomised based on the recommended target value of >50 nmol/L [2], and categorised into groups of <27.5, 27.5 to <50, 50 to <78, and ≥78 nmol/L. A country of origin group was created where country of origin was categorised into South Asia, Central Asia, Central Africa, East Africa, North Africa or Middle East. Actual age in years at each visit was calculated by subtracting the date of birth from the date of assessment. For all analyses, 95% confidence intervals (CI) are reported and significance was set at *p* < 0.05.

At each time point, demographic, anthropometric data (age, height and weight), biochemical parameters and sun exposure data were collected. Shapiro–Wilk test result was used to assess the assumption of normal distribution. The treatment group differences were examined using independent *t*-test (or the non-parametric alternative Mann–Whitney U). At each time point, gender, season and dichotomised 25(OH)D treatment group differences were examined using Chi Square, reporting Fisher’s Exact test results for cell counts less than five.

Three longitudinal investigations were conducted using a Generalized Linear Mixed Model (GLMM). GLMM are an extension of the flexible linear mixed model but incorporate random effects which are useful for accommodating the heterogeneity present in repeated measures (longitudinal design), and ability to also model count and binary data [29]. GLMM was used to examine the longitudinal changes in 25(OH)D over time examining time (weeks) and treatment group while controlling for age, gender, country of origin (grouped), calcium, ALP, season and sun exposure score. Individuals were set as a random effect. Model 1 investigated 25(OH)D as a scale variable using a linear GLMM which allowed for each variables’ contribution to changes in serum 25(OH)D to be quantified. Model 2 and model 3 investigated 25(OH)D as a dichotomous variable (>50 and ≥78 nmol/L respectively) using a binary logistic regression GLMM which allowed for each variables’ contribution to be quantified with respect to risk of having 25(OH)D serum levels above the nominated cut-off.

## 3. Results

**Figure 2:**
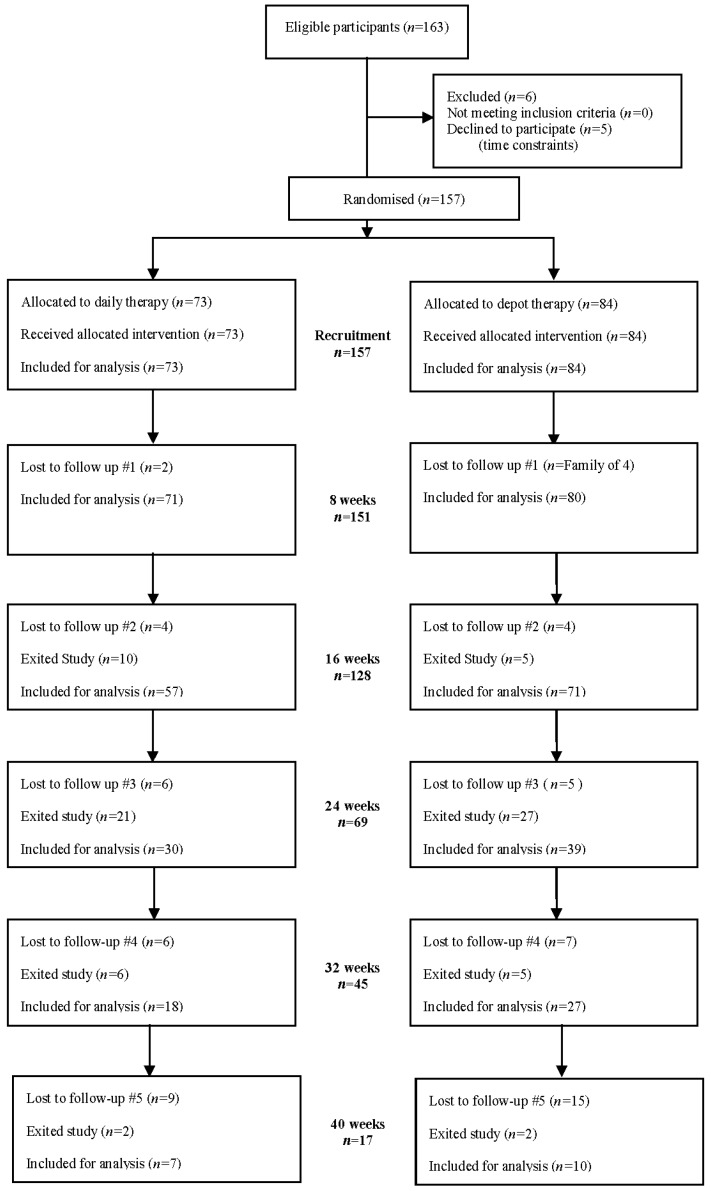
CONSORT flow diagram.

The flow of participants is described according to CONSORT criteria (Figure 2). Of the 163 eligible participants, 157 participants (96.3%) were recruited and randomised to daily (*n* = 73) or depot therapy (*n* = 84) at baseline. Six participants did not return after the initial baseline visit and were not included in the data analyses. The final sample for analysis was 151 (92.6%), with 71 receiving daily and 80 receiving depot therapy.

### 3.1. Baseline Characteristics of the Study Population

Participants of the study ranged in age from 5.5 months to 16 years, with participants in the daily treatment group (median = 8.05) slightly older than the depot treatment group (median = 7.49; *p* = 0.023). Both groups were similar with respect to demographic and anthropometric parameters indicating that the two groups were comparable at baseline (*p* > 0.05) (Table 1).

Most of the influx of refugees were from Central Asia (25.8%), then South Asia (23.2%) with no treatment group differences (χ^2^ = 8.08 *p* = 0.151). Of these, 29.8% transited through South Asia (34.4%) with no treatment group differences (χ^2^ = 1.14 *p* = 0.904).

The majority of participants were recruited for the study during the season of winter (*n* = 85, 56.3%), the least in summer (*n* = 7, 4.6%). There were no seasonal differences at baseline between treatment groups (χ^2^ = 1.70 *p* = 0.668).

### 3.2. Clinical Findings

None of the participants had clinical signs of rickets throughout the study.

### 3.3. Biochemical Parameters and Surrounding Factors

**Figure 3:**
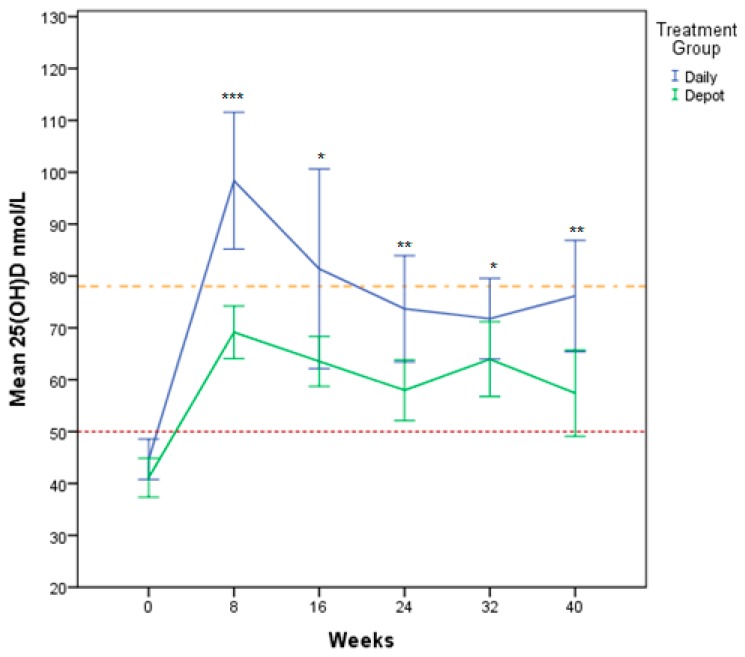
Mean 25(OH)D (nmol/L) change over time for Daily and Depot Treatment Groups. Raw mean 25(OH)D nmol/L change over time. -- represents threshold level 50 nmol/L -- represents threshold level 78 nmol/L * *p* < 0.05; **p < 0.01; *** *p* < 0.001.

Biochemical and sun exposure data are described in Table 2. The daily treatment group had significantly higher 25(OH)D levels at each visit post baseline (Figure 3). The dichotomised 25(OH)D variable showed that the daily treatment group had a higher proportion of participants with levels above 50 nmol/L at all time points, however this was not statistically significant at any time point (Table 3).

One participant (eight months of age, breastfed with introduction of solids, 7.9 kg at eight weeks of treatment; 10 months, 9 kg at 16 weeks of treatment) had intermittent hypervitaminosis D on daily therapy with 25(OH)D level of 440 and 595 nmol/L at 8 and 16 weeks respectively. The calcium levels were 2.70 mmol/L (2.15–2.65 mmol/L) and 2.31 mmol/L respectively. The participant was asymptomatic. The follow up level was 71 nmol/L after 14 weeks.

Calcium and ALP levels were similar for both treatment groups at each time point (Table 2) and were within normal range (Ca 2.15–2.65 mmol/L, ALP ≤ 420 U/L). However, three participants presented with asymptomatic hypocalcaemia (<2.15 mmol/L) at recruitment. Albumin adjusted calcium levels were 2.02 mmol/L, 2.09 mmol/L and 2.04 mmol/L; 25(OH)D 47 nmol/L, 34 nmol/L and 40 nmol/L; ALP 385 U/L, 174 U/L and 206 U/L respectively. One participant presented with asymptomatic hypercalcaemia of 2.7 mmol/L at their eight weeks follow up.

Sun exposure scores were similar at baseline, but differentiated significantly between treatment groups at 24, 32 and 40 weeks, with the daily treatment group reporting higher scores.

**Figure 4:**
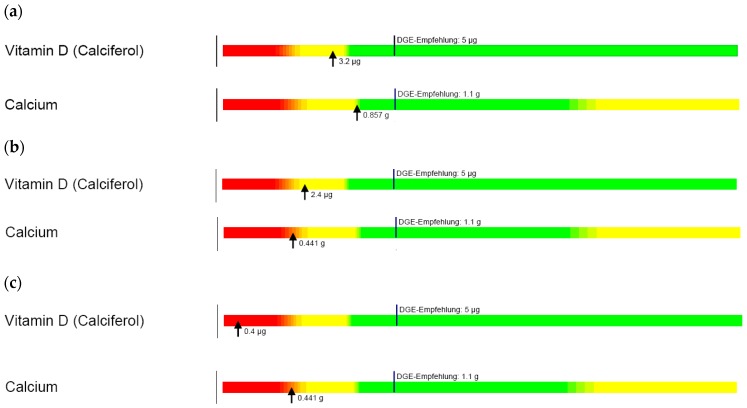
Typical dietary vitamin D and calcium intakes in (**a**) African; (**b**) Middle-Eastern and (**c**) Asian families. (Arrows indicate the actual intake, while vertical lines represent the recommended daily intake). DGE empfehlung = German Nutrition Society (DGE) recommendation

Results of the dietary analysis are shown in Figure 4. Typical diets of African families consisted of bread, rice, lamb, beef, chicken, pasta and various vegetables with 1–3 cups of milk per day. The typical diet of Islamic/Middle-Eastern families consisted of bread, potato, chicken, fish, beef, lamb, vegetables and pasta with 0–2 cups of milk daily, while in Asian families noodles, rice, chicken, beef, eggs, pork and vegetables were common with 0–1 cups of milk per day. The analyses revealed that none of these groups obtained the recommended dietary intake of calcium or vitamin D.

**Figure 5:**
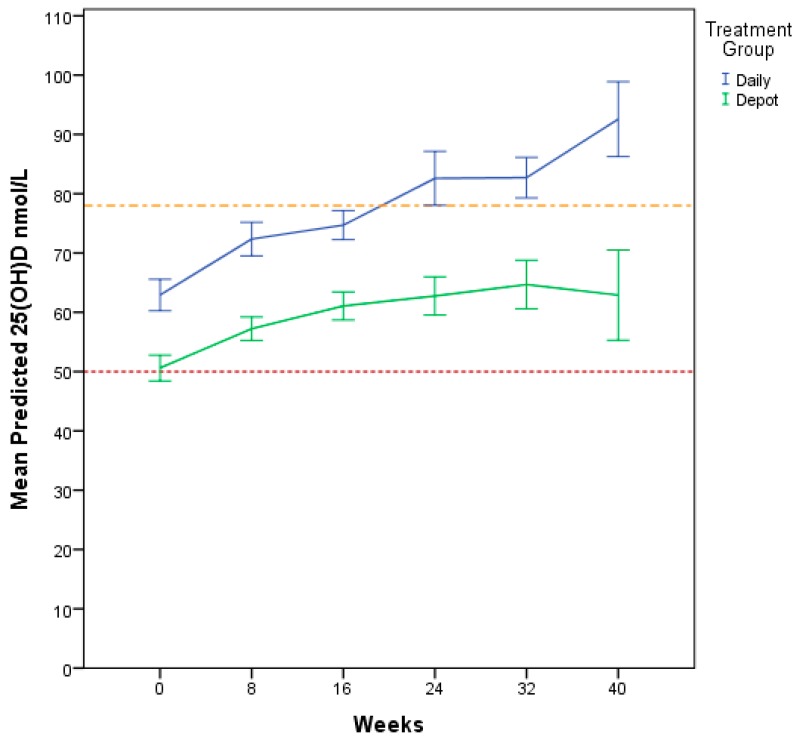
Generalized Linear Mixed Models (GLMM) Model 1 Predicted mean 25(OH)D nmol/L change over time; - - 50 nmol/L threshold - - 78 nmol/L threshold.

The GLMM for Model 1 (Table 4) revealed time (*p* < 0.001), sun exposure score (*p* = 0.046), treatment group (*p* < 0.001), and calcium (*p* < 0.001) as significant predictors of 25(OH)D serum levels. Vitamin 25(OH)D serum levels increased over time (β = 4.80, 95% CI 2.47–7.14), and were higher for the daily treatment group (β = 13.59, 95% CI 7.39–19.79). When accounting for contributing factors (Model 1) the predicted 25(OH)D serum levels over time show demonstrated better improvement pattern for participants in the daily treatment group (Figure 5).

The GLMM for Model 2 (Table 5) reported duration of treatment and season as significant predictor for the dichotomous 25(OH)D serum levels (*p* < 0.001). However, of clinical interest was the participant’s odds (OR) of having 25(OH)D serum levels above 50 nmol/L. Participants were more likely to reach this threshold if they received the daily treatment (OR = 1.5, 95% CI 0.9–2.7), had higher calcium levels (OR = 3.6, 95% CI 0.2–73.5), had higher sun exposure scores (OR = 11.1 95% CI 0.5–226.7), and if the participant was from Central Africa (OR = 2.4, 95% CI 0.6–9.4) or North Africa (OR = 2.0, 95% CI 0.6–6.3) compared to participants from South Asia. In summer, the likelihood of achieving Vitamin D sufficiency was 2.3 times (95% CI 1.1–4.8) compared to winter (*p* = 0.034). When investigating 25(OH)D serum levels ≥above 78 nmol/L (Table 6) treatment group, time, age, season, sun exposure and country of origin remained important risk modifiers.

## 4. Discussion

This is a randomised controlled clinical trial comparing the outcome of daily versus depot vitamin D supplementation in a group of newly-settled refugee children and adolescents in Western Australia. Participants on both daily and depot regimens achieved vitamin D sufficiency (Figure 3). The therapeutic outcome was influenced by time, sun exposure score, calcium and treatment group. Studies looking at vitamin D status in recently arrived immigrants confirm similar patterns with highest prevalence of vitamin D deficiency in Middle Eastern populations [30,31]. This is of growing global importance in the context of the ongoing Syrian refugee crisis and resettlement health challenges internationally.

Our study demonstrated statistical difference between treatment groups for 25(OH)D at all follow up time points with higher serum levels for the daily group. Predictive modelling controlling for other determining factors supported this relationship, indicating that daily treatment was 1.5 times more likely to improve and maintain vitamin D levels. The drop in vitamin D levels for the daily treatment group after eight weeks is most likely related to a lack of adherence to treatment. With longer duration of treatment, it is possible that only the most dedicated participants showing high adherence to daily treatment regimen are remaining. This consistent decline could be due to a change in patient behaviour resulting from their involvement in a study known as the “Hawthorne effect” [32,33,34]. It is also possible that this outcome was affected by the fact that the depot dose chosen was slightly lower than the potential cumulative dose for the daily doses i.e., the 5000 IU/day group would have had 280,000 IU cumulative dose (5000 IU/day × 56 days) between visits compared to the 200,000 IU given as a single depot dose. Similarly, the 2500 IU/day group received 140,000 IU cumulative dose (2500 IU × 56 days) between visits compared to the 100,000 IU given as a depot dose. We were conservative with the calculation of the depot dose as we could not include an early follow up to analyse for peak values post depot administration. A second rationale was that this would allow compensating for non-adherence in the daily group. Importantly, the study demonstrates that even with treatment at the relatively lower depot vitamin D dose, 25(OH)D levels were sufficient. For the maintenance phase, the depot dose was higher than the potential sum of the daily doses (35,000 versus 22,400 IU). This regimen allowed further stabilisation of 25 OHD levels.

Several studies have used depot or stoss therapy or daily treatment to treat vitamin D deficiency. Many of the studies are adult studies but there are increasing numbers of studies in children (Supplementary Table S1), but study/trial designs are significantly heterogenous, making direct comparisons difficult. Importantly the variability in vitamin D replacement regimens with respect to formulation (Ergocalciferol, (vitamin D2) or Cholecalciferol (vitamin D3)), dosing (low dose 1000–5000 IU or depot 150,000–600,000 IU), frequency (daily, weekly/bi-weekly and stat/intermittent) and different routes of delivery (intramuscular or oral) may influence generalisability of results to other clinical settings.

The majority of the trials in children have used single high doses of vitamin D [35,36,37,38,39,40] or compared different doses or duration of depot preparations [41,42,43,44,45,46,47,48,49,50] for treatment. There are only limited numbers of trials comparing low dose daily against intermittent depot vitamin D therapy [51,52,53,54,55,56,57,58]. Studies using depot vitamin D treatment in children suggest that high-dose repletion approaches are safe and effective. Various combinations of high doses have been used including 600,000 IU vitamin D2 [36], 60,000 IU vitamin D3 weekly for 4–8 weeks [41], intermittent 50,000 IU to 300,000 IU 1–3 monthly over 12 months [59], 600,000 IU single dose versus 60,000 IU weekly over eight weeks [46], 45,000 IU weekly for two months [57], 100,000 IU bimonthly (total three doses) during winter [60] and were reported to be effective with no hypercalcaemia, hypercalcuria or nephrocalcinosis observed. Cesur et al. [45] compared depot vitamin D doses of 150,000 IU, 300,000 IU and 600,000 IU to treat vitamin D deficiency rickets in children 3–36 months of age and showed no differences in the improvement of rickets between the different doses, but two of the 300,000 IU group (*n* = 16) and six of the 600,000 IU group (*n* = 16) developed hypercalcaemia. In our cohort asymptomatic hypercalcaemia was seen in only one child in the daily arm at eight weeks. Similarly, hypercalcaemia and/or hypercalciuria has been reported when 300,000 IU or 600,000 IU of an oral depot solution is used [47,61]. Severe hypercalcaemia was not seen in a cohort of 987 infants supplemented 400 IU to 1200 IU daily at 6 and 12 months [62]. Interestingly, in a study by Vijayakumar and Meenu [46] comparing effectiveness of 600,000 IU stat versus 60,000 IU weekly oral dosing in children with nutritional rickets; calcium supplementation was administered to all children for 12 months without any demonstrated increased risk of hypercalcaemia.

Some literature exists exploring safety and efficacy of comparative treatment regimens. Specifically, daily treatment (2000 IU D3 for six weeks [51,55,56], 2000 IU D2 [55,56] and 20 days [52], 400 IU D3 or 2000 IU D3 for two months then 1000 IU D3 daily over 12 months [57]) against depot treatment (50,000 IU D2 weekly for six weeks [55,56], 150,000 IU D3 [51] and 600,000 IU D3 [52], 45,000 IU D3 weekly for two months then 400 IU D3 daily [57]). Vitamin D level post treatment in depot therapy groups were significantly higher compared to daily vitamin D treatment groups [51,52]. No difference was seen in the other two studies [55,56]. Talaat et al. [57], randomised clinically asymptomatic vitamin D deficient children aged 2 to 18 years into three different vitamin D3 replacement regimens: 400 IU daily or 45,000 IU weekly for two months then 400 IU daily or 2000 IU daily for three months then 1000 IU daily for a period of 12 months with assessment at the 4 and 12 month time points. No dosing adjustments were made to vitamin D therapy at follow up assessment. Similar to our study, daily replacement therapy was the best therapeutic regimen in maintaining sufficient vitamin D levels.

A recent global consensus statement on nutritional rickets [2] defines vitamin D toxicity as hypercalcaemia and 25(OH)D >250 nmol/L with hypercalciuria and suppressed PTH. These cases are regarded as being rare and usually asymptomatic, limited data exists exploring toxic doses of vitamin D [63]. In infants and young children, toxicity has been reported after dosage in the range of 240,000 IU to 4,500,000 IU [63]. Only one participant in our study, receiving daily supplementation, had hypervitaminosis D and hypercalcaemia of 2.70 mmol/L but remained asymptomatic. We ensured the participant was being given the correct dose and not excess supplementation. Unfortunately, a renal ultrasound was not performed; levels normalised on repeat testing at 14 weeks.

Studies looking at safety of depot vitamin D therapy have not reported significant evidence of hypercalcaemia or renal nephrocalcinosis, but follow-up periods were predominantly <8 weeks. Compared to our study, relatively higher doses of depot treatment [52] were used and in two of the studies, vitamin D2 rather than vitamin D3 was used. Our cohort provides longitudinal data to 40 weeks, which is a key strength of this study. Talaat et al. [57] reported calcium and hypercalciuria at 4 months and found up to 1.5% of cases hypercalciuria in the daily replacement group and no reported hypercalcaemia in any of the participants. It is postulated that daily or weekly therapy are more physiological and effective with less adverse effects compared to depot therapy [22,64]. However, depot therapy is a preferred option when adherence to therapy is uncertain [2].

None of our participants had clinical signs of rickets; routine radiological imaging was not undertaken. Similarly, in a study by Jain et al. [65], none of the 98 infants (aged 2.5–3.5 months) with 25(OH)D <25 nmol/L showed any clinical signs of rickets. Studies by Ladhani et al. [66], Gordon et al. [67] and Perez-Rossello [68] included radiological assessment in addition to clinical assessment in children with vitamin D deficiency and reported radiological changes in 32.5% (all clinically asymptomatic) to 70% (no mention of clinical evidence of rickets) of the children with vitamin D deficiency. Given the varying aetiology and prevalence of rickets in infants, children and adolescents with vitamin D deficiency, clinical signs alone are not adequate for screening and diagnosis. The role of ALP as a screening parameter for vitamin D deficiency is controversial. ALP is an enzyme found in all body tissues, including osteoblasts where it is a marker for bone formation. It is especially utilised as a bone marker due to being cost effective. Whilst levels of ALP have been noted to raise during vitamin D deficiency, thought to be due to regeneration of bone tissue, it is not a suitable marker for children due to its wide distribution and increase during skeletal growth [69,70]. ALP levels vary according to age, race and gender, making it difficult to associate vitamin D deficiency with ALP levels alone [71,72,73,74]. In our study clinical findings and ALP were not reliable markers of vitamin D deficiency in line with other studies [70]. We did not measure bone specific ALP, which has been shown to be a reliable marker [18]. Population-based screening with serum 25(OH)D, ALP and radiographs is not recommended [2]. However, testing serum 25(OH)D in at risk populations can be considered.

Depot therapy has been proposed as an alternative to daily therapy to improve the therapeutic outcome. In the Global Consensus paper daily therapy was preferred over depot therapy acknowledging that depot therapy may be more practical in certain situations such as non-adherence to treatment [2]. An important factor in the context is that depot therapy can be administered under supervision in clinic. A successful example for this strategy is “directly observed therapy for treating tuberculosis” [75]. Depot utilisation in more vulnerable clinical cohorts (e.g., young children, adolescents, limited English proficiency or itinerant populations) also needs consideration.

Significant predictors of 25(OH)D serum levels were time, treatment group, calcium and sun exposure score. Gender, age, season and country of refuge/origin did not significantly influence the treatment effectiveness depending on chosen cut-off points. A significant positive change in sun exposure scores at each of the follow up visits was observed in the daily treatment group compared to the depot group, with statistically significant group differences at 24, 32 and 40 weeks. It is likely that taking daily treatment is a reminder of need for sun exposure as opposed to depot treatment. A positive association between sun exposure and 25(OH)D3 levels has been confirmed in a few studies by Jones et al. [26,76], although this association became non-significant once adjusted for number of sports played [26]. Unfortunately, in our study, sport participation information was not obtained.

Dietary analysis confirmed that traditional diets of all families, regardless of ethnic background, were deficient in calcium and vitamin D. Newly settled migrants and refugees are often in poor health due to prolonged periods in their country of origin or refuge before entering Australia [5,77]. Once in Australia, unfamiliarity with local produce, food insecurity, accessibility, limited health literacy, language barriers and acculturation (e.g., consumption of high-fat, high-calorie fast foods) can further influence suboptimal nutrition [5,77,78,79]. Our data demonstrate the importance of nutritional assessment for refugee children and provision of culturally appropriate health and nutrition education to allow families to familiarise themselves with local produce and foods and optimise nutritional intake in keeping with age-appropriate recommendations [79].

There were limitations to this study design. This study focussed on prospective longitudinal follow up of vitamin D treatment using different treatment modes. As such, one limitation is the lack of data regarding potential vitamin D toxicity at the end of the initial two weeks following depot treatment as there was no follow up at this time point.

It should be noted that the study was underpowered statistically at 40 weeks for the daily group, although statistical significance was still shown, suggesting sufficient power. Post study design and implementation, it was decided to analyse four categories of Vitamin D, hence the Chi square analysis is likely underpowered for detecting significant group differences, but data suggests that there is a difference between treatment groups and Vitamin D categories that should be further explored in future research studies.

Loss to follow-up was significant, however engagement through the paediatric RHS facilitated longitudinal follow-up, particularly in a mobile and predominantly non-English proficient cohort. No comparative international data currently exist beyond a period post-treatment; our data provide important evidence to improve this gap in knowledge. Importantly, sample selection bias was negated by the recruitment through the centralised refugee screening service.

Different study preparations of vitamin D solutions were utilised; the daily regimen arm received a commercially available preparation whilst the depot D3 therapy was prepared by the Clinical Trials Pharmacy. The depot solution required refrigeration and any precipitation may have altered the concentration of the solution, although precautions were in place in the study protocol with respect to time for warming prior to administration. Consequently, the solution half-lives of the solutions may have differed however the high number of participants and long duration of the study is likely to have reduced this variability.

Compliance with the daily vitamin D supplementation was assessed by parental report via interpreters, but formal review of bottles was not undertaken. Similarly, a degree of recall bias may have influenced sun exposure reporting and dietary questionnaires. Interestingly, vitamin D intoxication was only noted in the daily treatment group at the scheduled visits. It can be speculated that this risk of overdosing on daily therapy needs to be considered as a factor in favour of depot therapy when depot therapy under supervision represents a safe alternative.

## 5. Conclusions

Our study provides important clinical data demonstrating the efficacy of depot vitamin D therapy as an alternative to daily supplementation in refugee children and adolescents both in short and longer-term follow-up durations. Use of depot formulations may be beneficial to ensure compliance in higher risk cohorts, particularly where daily administration may be challenging. Supplementation of vitamin D, needs to be combined by improved nutritional assessment and culturally sensitive education programmes following resettlement to ensure that longer term dietary requirements are being addressed.

## Figures and Tables

**Table 1:** Baseline characteristics of study population.

	Vitamin D3 Therapy						
	Daily Treatment			Depot Treatment			Daily Vs. Depot *p*-Value
	*n*	Mean (SD)	95% CI	*n*	Mean (SD)	95% CI	
Age (years)	71	8.05 (4.29)	7.04–9.07	80	7.49 (4.23)	6.54–8.43	0.023 *
Height (cm)	64	124.6 (26.2)	118.0–131.1	75	120.5 (26.1)	114.5–126.5	0.317
Weight (kg)	67	28.0 (16.0)	24.1–32.0	76	27.2 (14.7)	23.9–30.6	0.772
Gender		37 male, 34 female			36 male, 44 female		χ^2^ = 2.21, *p* = 0.137

Note: * Indicates significant Independent Samples Mann–Whitney U Test for treatment group differences (*p* < 0.05). SD = Standard Deviation.

**Table 2:** Biochemical and sun exposure data.

		Vitamin D_3_ Treatment		Daily Vs. Depot *p*-Value
	Normal Range	Daily Mean (95% CI)	Depot Mean (95% CI)	
**25(OH)D (nmol/L)**				
Baseline (*n* = 71; 80)		45 (41–49)	41 (37–45)	0.223
8 weeks (*n* = 71; 79)		98 (85–112)	69 (64–74)	<0.001 *
16 weeks (*n* = 57; 71)		81 (62–101)	64 (59–68)	0.021 *
24 weeks (*n* =30; 39)		74 (63–84)	58 (52–64)	0.005 *
32 weeks (*n* = 18; 27)		72 (64–80)	64 (57–71)	0.032
40 weeks (*n* = 7; 10)		76 (65–87)	57 (49–66)	0.005 ^a,^*
**Calcium (mmol/L)**	1.90–2.60			
Baseline (*n* = 69; 80)		2.35 (2.33–2.37)	2.34 (2.32–2.36)	0.571 ^a^
8 weeks (*n* = 71; 78)		2.36 (2.34–2.38)	2.35 (2.33–2.37)	0.361
16 weeks (*n* = 56; 67)		2.33 (2.31–2.35)	2.33 (2.32–2.35)	0.876 ^a^
24 weeks (*n* = 27; 36)		2.33 (2.30–2.36)	2.31 (2.28–2.33)	0.163 ^a^
32 weeks (*n* = 18; 24)		2.30 (2.28–2.33)	2.28 (2.24–2.31)	0.231 ^a^
40 weeks (*n* = 7; 10)		2.28 (2.24–2.33)	2.26 (2.22–2.30)	0.367 ^a^
**ALP (U/L)**	40–420			
Baseline (*n* = 68; 80)		257 (233–280)	253 (229–276)	0.669
8 weeks (*n* = 71; 78)		275 (250–300)	255 (233–278)	0.230
16 weeks (*n* = 55; 64)		258 (235–281)	240 (214–264)	0.264
24 weeks (*n* = 28; 34)		270 (233–307)	253 (212–294)	0.369
32 weeks (*n* = 17; 24)		275 (230–319)	255 (208–303)	0.435
40 weeks (*n* = 7; 10)		247 (191–303)	254 (162–345)	0.904 ^a^
**Sun exposure Score**	-			
Baseline (*n* = 71; 79)		0.10 (0.08–0.12)	0.10 (0.08–0.13)	0.398
8 weeks (*n* = 71; 80)		0.14 (0.11–0.16)	0.10 (0.09–0.12)	0.158
16 weeks (*n* = 57; 71)		0.13 (0.11–0.16)	0.12 (0.11–0.14)	0.996
24 weeks (*n* = 30; 39)		0.20 (0.16–0.25)	0.12 (0.09–0.14)	0.005 *
32 weeks (*n* = 18; 27)		0.20 (0.16–0.25)	0.11 (0.08–0.13)	0.001 *
40 weeks (*n* = 7; 9)		0.25 (0.24–0.26)	0.11 (0.07–0.15)	<0.001 *

Note: * Indicates significant treatment group differences (*p* < 0.05); ^a^ Indicates an independent *t*-test was used, otherwise the non-parametric alternative Mann–Whitney test result is reported.

**Table 3:** Frequency (percent) of participants according to 25(OH)D level cut-off points.

nmol/L	Vitamin D3 Treatment								χ_2_ (*p*-Value)
	Daily				Depot				
	<27.5	27.5 to <50	50 to <78	≥78	<27.5	27.5 to <50	50 to <78	≥78	
Baseline (*n* = 71; 80)	15 (21.1%)	22 (31.0%)	34 (47.9%)	0 (0%)	19 (23.8%)	31 (38.8%)	30 (37.5%)	0 (0%)	1.72 (*p* = 0.440)
8 weeks (*n* = 71; 79)	0 (0%)	5 (7.0%)	25 (35.2%)	41 (57.7%)	1 (1.3%)	14 (17.7%)	35 (44.3%)	29 (36.7%)	8.44 (*p* = 0.023) ^a,^*
16 weeks (*n* = 57; 71)	0 (0%)	10 (17.5%)	23 (40.4%)	24 (42.1%)	1 (1.4%)	17 (23.9%)	38 (53.5%)	15 (21.1%)	6.96 (*p* = 0.050) ^a^
24 weeks (*n* =30; 39)	0 (0%)	4 (13.3%)	16 (53.3%)	10 (33.3%)	2 (5.1%)	11 (28.2%)	22 (56.4%)	4 (10.3%)	7.15 (*p* = 0.048) ^a,^*
32 weeks (*n* = 18; 27)	0 (0%)	1 (5.6%)	13 (72.2%)	4 (22.2%)	0 (0%)	5 (18.5%)	19 (70.4%)	3 (11.1%)	2.10 (*p* = 0.451) ^a^
40 weeks (*n* = 7; 10)	0 (0%)	0 (0%)	5 (71.4%)	2 (28.6%)	0 (0%)	2 (20.0%)	7 (70.0%)	1 (10.0%)	1.92 (*p* = 0.460) ^a^

^a^ Fisher’s Exact Chi square results are reported; * Indicates statistical significance *p* < 0.05.

**Table 4:** Generalized Linear Mixed Models (GLMM) Model 1 Parameter Estimates for Vitamin D serum levels (25(OH)D nmol/L).

Model Term	β Coefficient	SE	*p*-Value	95% CI	
				Lower	Upper
Intercept	−103.06	42.61	0.016	−186.77	−19.35
Time *	4.80	1.19	<0.001	2.47	7.14
Female	−2.58	3.01	0.391	−8.49	3.33
Male (reference)	0				
Age	−0.72	0.38	0.060	−1.47	0.03
Season = Autumn	−8.28	4.58	0.071	−17.28	0.71
Season = Spring	3.80	3.32	0.253	−2.73	10.33
Season = Summer	0.32	4.42	0.943	−8.36	9.00
Season = Winter (reference)	0				
Daily Treatment Group *	13.59	3.16	<0.001	7.39	19.79
Depot (reference)	0				
Calcium *	67.13	17.47	<0.001	32.81	101.44
ALP	−0.02	0.02	0.315	−0.05	0.01
Sun Exposure Score *	33.29	16.63	0.046	0.63	65.95
Country of Origin group = Central Africa	−7.31	6.94	0.293	−20.96	6.33
Country of Origin group = East Africa	−1.47	4.97	0.767	−11.23	8.28
Country of Origin group = North Africa	2.76	5.78	0.633	−8.60	14.11
Country of Origin group = Middle East	−6.11	4.69	0.194	−15.33	3.12
Country of Origin group = Central Asia	2.63	4.29	0.540	−5.79	11.06
Country of Origin group = South Asia (reference)	0				

* Indicates significance (*p* < 0.05); SE = Standard Error.

**Table 5:** GLMM Model 2 Parameter Estimates for meeting Vitamin D serum threshold (25(OH)D >50 nmol/L).

Model Term	Coefficient	SE	*p*-Value	95% CI		OR	95% CI OR	
				Lower	Upper		Lower	Upper
Time (weeks) *	0.52	0.11	<0.001	0.30	0.73	1.68	1.35	2.08
Female	−0.05	0.28	0.861	−0.60	0.50	0.95	0.55	1.65
Male (reference)	0							
Age	−0.01	0.04	0.752	−0.08	0.06	0.99	0.92	1.06
Season = Autumn	0.45	0.37	0.224	−0.28	1.18	1.57	0.76	3.25
Season = Spring	0.54	0.28	0.054	−0.01	1.08	1.71	0.99	2.94
Season = Summer *	0.82	0.39	0.034	0.06	1.58	2.27	1.06	4.84
Season = Winter (reference)	0							
Daily Treatment Group	0.42	0.29	0.149	−0.15	0.99	1.52	0.86	2.68
Depot (reference)	0							
Calcium	1.29	1.53	0.398	−1.71	4.30	3.65	0.18	73.46
ALP	−0.01	0.00	0.286	−0.04	0.01	1.00	1.00	1.00
Sun Exposure Score	2.41	1.53	0.117	−0.60	5.42	11.14	0.55	226.65
Country of Origin group = Central Africa	0.86	0.71	0.225	−0.53	2.24	2.36	0.59	9.43
Country of Origin group = East Africa	−0.20	0.45	0.653	−1.09	0.68	0.82	0.34	1.98
Country of Origin group = North Africa	0.70	0.58	0.229	−0.44	1.84	2.01	0.64	6.28
Country of Origin group = Middle East	−0.63	0.42	0.138	−1.46	0.20	0.53	0.23	1.22
Country of Origin group = Central Asia	−0.07	0.40	0.863	−0.85	0.71	0.93	0.43	2.03
Country of Origin group = South Asia (reference)	0							

SE = standard error; OR = odds ratio; * Indicates significance (*p* < 0.05). Shaded grey indicates clinically relevant risk factors.

**Table 6:** GLMM Model 3 Parameter Estimates for meeting Vitamin D serum threshold (25(OH)D ≥78 nmol/L).

Model Term	Coefficient	SE	*p*-Value	95% CI		OR	95% CI OR	
				Lower	Upper		Lower	Upper
Time (weeks) *	0.24	0.11	0.036	0.02	0.46	1.27	1.02	1.58
Female	0.31	0.25	0.222	−0.19	0.80	1.36	0.83	2.23
Male (reference)	0							
Age *	−0.09	0.03	0.010	−0.15	−0.02	0.92	0.86	0.98
Season = Autumn *	−1.82	0.65	0.006	−3.10	−0.53	0.16	0.05	0.59
Season = Spring	0.38	0.29	0.192	−0.19	0.95	1.46	0.83	2.58
Season = Summer	−0.41	0.41	0.321	−1.22	0.40	0.67	0.30	1.49
Season = Winter (reference)	0							
Daily Treatment Group *	0.81	0.27	0.003	0.28	1.34	2.24	1.32	3.82
Depot (reference)	0							
Calcium	2.68	1.55	0.085	−0.37	5.73	14.54	0.69	306.62
ALP	−0.00	0.00	0.599	−0.00	0.00	1.00	1.00	1.00
Sun Exposure Score *	3.06	1.36	0.025	0.39	5.72	21.28	1.48	305.40
Country of Origin group = Central Africa *	−1.41	0.65	0.031	−2.68	−0.13	0.25	0.07	0.88
Country of Origin group = East Africa	−0.30	0.41	0.465	−1.10	0.50	0.74	0.33	1.66
Country of Origin group = North Africa	0.76	0.43	0.076	−0.08	1.61	2.14	0.92	4.98
Country of Origin group = Middle East	−0.47	0.40	0.241	−1.26	0.32	0.63	0.29	1.37
Country of Origin group = Central Asia	−0.25	0.36	0.491	−0.96	0.46	0.78	0.38	1.59
Country of Origin group = South Asia (reference)	0	.	.	.	.	.	.	.

SE = standard error. OR = odds ratio. * indicates significance (*p* < 0.05). Shaded grey indicates clinically relevant risk factors.

## Supplementary Materials

The following are available online at http://www.mdpi.com/2072-6643/10/3/348/s1, Table S1: Summary of paediatric published literature looking at oral depot and/or daily vitamin D therapy.
